# Effect of Diet on Expression of Genes Involved in Lipid Metabolism, Oxidative Stress, and Inflammation in Mouse Liver–Insights into Mechanisms of Hepatic Steatosis

**DOI:** 10.1371/journal.pone.0088584

**Published:** 2014-02-14

**Authors:** Helen J. Renaud, Julia Y. Cui, Hong Lu, Curtis D. Klaassen

**Affiliations:** 1 Department of Internal Medicine, Kansas University Medical Center, Kansas City, Kansas, United States of America; 2 Department of Pharmacology, State University of New York, Syracuse, New York, United States of America; University of Texas Health Science Center at San Antonio, United States of America

## Abstract

Nutritional intake is a fundamental determinant of health. Many studies have correlated excess caloric intake, as well as a high ratio of n-6:n-3 fatty acids, with detrimental health outcomes, such as the metabolic syndrome. In contrast, low-calorie diets have beneficial health effects. Despite these associations, our understanding of the causal relationship between diet and health remains largely elusive. The present study examined the molecular changes elicited by nine diets with varying fat, sugar, cholesterol, omega-3 fatty acids, omega-6 fatty acids, and calories in C57BL/6 male mice. Microarray analyses were conducted on liver samples from three mice per diet and detected 20,449 genes of which 3,734 were responsive to changes in dietary components. Principal component analysis showed that diet restriction correlated the least with the other diets and also affected more genes than any other diet. Interestingly, Gene Set Enrichment Analysis (GSEA) identified gene sets involved in glutathione metabolism, immune response, fatty acid metabolism, cholesterol metabolism, ABC transporters, and oxidative phosphorylation as being highly responsive to changes in diet composition. On the gene level, this study reveals novel findings such as the induction of the drug efflux pump Abcb1a (p-glycoprotein) by diet restriction and an atherogenic diet, as well as the suppression of the rate limiting step of bile acid synthesis, Cyp7a1, by a high fructose diet. This study provides considerable insight into the molecular changes incurred by a variety of diets and furthers our understanding of the causal relationships between diet and health.

## Introduction

Diet plays an influential role in the maintenance of human health and the progression of pathologic states. Diets high in saturated fats, sugars, and with high ratios of n-6:n-3 polyunsaturated fatty acids (PUFAs) are associated with development of the metabolic syndrome in humans [1], [2], [3], [4], as well as rats and mice [5], [6]. In contrast, diets consisting of low caloric intake alleviate symptoms of the metabolic syndrome in humans [7] and mice [8]. The metabolic syndrome is generally characterized by a constellation of abnormalities including fasting hyperglycemia, dyslipidemia (high triglycerides and low HDL cholesterol), central obesity, and high blood pressure. The clustering of these abnormalities increases the risk of developing type 2 diabetes, cardiovascular disease, non-alcoholic fatty liver disease (NAFLD), and certain cancers such as liver, colon, bladder, pancreatic, prostate, endometrial, and postmenopausal breast cancers [9], [10]. The incidence of the metabolic syndrome is increasing in the United States and it is estimated that 34% of Americans are now affected with this syndrome, up from 29% in 1994 [11].

NAFLD is recognized as the hepatic manifestation of the metabolic syndrome [12], and is characterized by the accumulation of fatty infiltrations affecting >5% of the liver [6], [13]. The clinical implications of NAFLD are derived by its potential to progress to steatohepatitis (NASH), fibrosis, cirrhosis, and in some cases, hepatocellular carcinoma [6], [14], [15]. The prevalence of NAFLD is rising in parallel with the increasing rate of obesity, and this global trend is attributed to the diet of the Western lifestyle [6]. The typical Western diet is generally characterized as containing excessive saturated fatty acids, insufficient n-3:n-6 PUFA ratios, and an excess amount of sugars [2].

The pathogenesis of NAFLD is not well understood, but is proposed to be a “two-hit” process [16]. The first “hit” leads to lipid accumulation and steatosis. Mechanisms of this lipid accumulation are unclear, but likely involve dysregulated lipid homeostasis including β-oxidation, very low density lipoprotein (VLDL) secretion, de novo lipogenesis, and lipid trafficking and storage [15]. This hepatic steatosis sensitizes the liver to a “second hit” leading to inflammation, a key pathophysiologic feature of steatohepatitis, and progressive liver disease [6], [16]. It has been proposed that the primary mediator of this “second hit” is oxidative stress [17], [18], [19].

Currently, the causal relationship between diet and liver pathogenesis is not well understood. To gain a better understanding of how diet influences the expression of hepatic genes that alter lipid metabolism, inflammation, and oxidative stress, the effect of diets containing varying amounts of cholesterol, fat, sucrose, fructose, and essential fatty acids (EFA, n-3 and n-6 fatty acids), and calories on hepatic gene expression in mice was determined in the present study.

## Methods and Materials

### Ethics Statement

The animal housing facility at the University of Kansas Medical Center is accredited by the Association for Assessment and Accreditation of Laboratory Animal Care. All procedures were approved by the University of Kansas Medical Center’s Institutional Animal Care and Use Committee.

### Animals

Ten-week old male C57BL/6 mice were purchased from Charles River Laboratories, Inc. (Wilmington, MA). Mice were housed in a temperature-, light-, and humidity-controlled environment. Mice (n = 5/diet) were fed one of the following diets (Harlan Laboratories) for 3 weeks in order to assess early diet-induced changes in hepatic gene expression (nutrient information for each diet is presented in **Table 1**):

**Table 1:** Macronutrients in each diet.

	AIN-93Purified	LabChow	HighFructose	HighFat	Western	Atherogenic	EFA Deficient	Low n-3
**Fat (g/kg)**	39.4	41	50	329	209	209	50	67.2
Saturated	6	9	19	151	136	136	50	7.32
MUFA	9.4	11	25	146	66	66	0	12.84
PUFA	24	21	6	32	7	7	0	43.34
n-6:n-3 PUFA	6.7	12	10.4	20.9	6	6	No n-3 or n-6	134.4
**Carbohydrates (dry weight g/kg)**	684	402	6–4	271	485	469	665	646
Sucrose (g/kg)	107	Very little	0	175	345.6	329.6	660.3	117.1
Fructose (g/kg)	0	Very little	600	0	0	0	0	0
Ploysaccharide (g/kg)	577	majority	4	96	139.4	139.4	4.7	528.9
**Cholesterol (mg/kg)**	28	50	89	54	2,077	13,077	8	40
**Protein (g/kg)**	124	243	183	239	173	173	176	190
**Cholic acid (g/kg)**	0	0	0	0	0	5	0	0
**Kcal/g**	3.6	3.0	3.6	5.1	4.5	4.5	3.8	3.8

1. TD.94048 - AIN-93M purified diet (control diet).

2. TD.8422 - Essential fatty acid (EFA) deficient diet.

3. TD.97070– High-fat diet.

4. TD.88137– High-saturated fat and -sucrose diet (Western diet).

5. TD.02028– High-fat, -sucrose, -cholesterol, and -cholic acid (CA) diet

(atherogenic diet).

6. TD.89247 - 60% fructose diet.

7. TD.8604– Natural ingredient diet (used in our animal facility) (Lab chow).

8. TD.00235+7% sunflower oil (low n-3 diet) [20].

9. Diet restriction - 75% of the diet TD.8604 consumed by *ad lib* feeding.

The *ad lib* daily feed intake was approximately 4 g/mouse. Therefore, for diet restriction, each mouse was given approximately 2.7–3.0 g of food per day. All mice were euthanized in the morning (8∶00–10∶00 A.M.) and blood and tissue samples were collected. Mice were not fasted before liver sample isolation.

### RNA Isolation

RNA was isolated using RNA-Bee RNA Isolation Reagent (Tel-Test Inc., Friendswood, TX) according to the manufacturer’s protocol. RNA concentrations were quantified using a NanoDrop1000 Spectrophotometer (NanoDrop Technologies, Wilmington, DE) at a wavelength of 260nm. RNA integrity was evaluated by an Agilent 2100 Bioanalyzer at the KUMC Microarray Facilities.

### Microarray

Gene expression in livers of mice treated with various diets was determined by the Affymetrix Mouse 430 2.0 Gene Expression arrays in the KUMC Microarray Core Facility. To ensure higher quality results in gene expression data from the microarray, we conducted microarrays on 3 mice per diet (27 microarrays in total) giving a biological n = 3 per diet, which lends statistical power to increase the confidence of the conclusions drawn from this experiment. Data were preprocessed by the GCRMA package in R and Bioconductor [21]. The expression values were in log_2_ scale. The gene annotations were generated using the annaffy package. Genes with average probe intensities above log_2_(100) in at least one diet were selected for follow-up analyses. The LIMMA package [22] was used to determine the differentially expressed genes among various diets, using the AIN-93M-purified diet as control (global method, false discovery rate adjusted, p<0.05). To determine statistical significance of data values from a gene symbol represented by multiple probes, values were averaged, and then a one-way ANOVA followed by a Dunnett’s multiple comparison test was performed (using AIN-93 purified diet as control). Microarray data was uploaded to the Gene Expression Omnibus database, accession number GSE51885.

### Pathway Analysis, Hierarchical Clustering, and Venn Diagrams

Pathway analysis was conducted using KEGG pathway gene sets in the GSEA software (Broad institute) [23]; only gene sets with FDR <25% were selected as a positive hit. One-Way hierarchical clustering was performed using the heatmap.2 function of the gplots package in R Bioconductor. Venn diagrams were calculated and illustrated using the vennCounts and vennDiagram functions of the Limma package in R Bioconductor.

### Principal Component Analysis

A principal component analysis was performed using GeneSpring version 11.5 (Agilent Technologies, Santa Clara, CA) to identify prevalent gene expression profiles affected by various dietary conditions. The score of each component is saved as expression profile, and the component loadings are saved for each gene for each component [24].

### RT-qPCR

Reverse transcription of RNA to cDNA was performed with the Applied Biosystems High Capacity Reverse Transcriptase kit (Applied Biosystems, Foster City, CA). Briefly, equal volumes of 50 ng/µl RNA and 2X reverse transcriptase and random primers reaction mix were combined and placed in an Eppendorf Mastercycler under the following conditions: 25°C for 10 min, 37°C for 120 min, and 85°C for 5 min. Subsequently, quantitative PCR (qPCR) was performed on the resulting cDNA. Primers for qPCR were designed with Primer-BLAST (www.ncbi.nlm.nih.gov/tools/primer-blast). All primers (Table S3) were synthesized by Integrated DNA Technologies (Coralville, IA). Briefly, the PCR reaction mix contained per reaction: 5 µl of Applied Biosystems SYBR® green PCR master mix, 2.5 µl of 3 µM forward and reverse primer mix, 0.5 µl RNAse-free H_2_O, and 2 µl of 2 ng/µl cDNA. Reactions were seeded in a 384-well optical reaction plate (Applied Biosystems) and fluorescence was quantified in real-time with the Applied Biosystems 7300 Real Time PCR System (Foster City, CA) under the following conditions: 50°C for 2 min, 95°C for 10 min, (95°C for 15 sec, 60°C for 1 min) X 40 cycles. Melt curves were conducted for every reaction. Relative mRNA expression was calculated by the comparative ΔΔCt method, using Gapdh as a reference transcript. Statistical differences in mRNA quantity between diets were determined with an unpaired, two-tailed Student’s t-test (when comparing two diets), or a one-way ANOVA followed by a Dunnet’s post-hoc test (when comparing more than two diets) using Graphpad Prism 4.0. Comparisons resulting with p<0.05 were considered statistically significant.

### Western Blot Analysis

Protein expression was examined utilizing western blotting. Approximately 50 mg of liver tissue was homogenized in 1 ml T-PER Tissue Protein Extraction Reagent (Thermo Scientific, Rockford, IL) +10 µl Protease Inhibitor Cocktail (Sigma, St. Louis, MO). Proteins were quantified spectrophotometrically using the BCA assay (Biorad, Hercules, CA). Protein (20 µg) was loaded into 12% acrylamide-containing SDS-PAGE gels, and transferred to PVDF membranes. Membranes were subsequently probed using antibodies specific for Cyp7a1 (1∶1000 dilution; ab65596; Abcam, Cambridge, MA) or α-tubulin (1∶5000 dilution; CP06; EMD-Millipore Corp, Billerica, MA). Following a one-hour incubation with species appropriate horse radish peroxidase-conjugated antibodies, membranes were immersed in Luminata Classico enhanced chemiluminescence reagent (EMD-Millipore Corp) followed by detection using GeneMate basic blue autoradiography film (Bioexpress, Kaysville, UT). Quantitative determination of Cyp7a1 band intensity was conducted by densitometry using ImageJ software [25]. Band intensity for each sample was normalized to the corresponding band intensity of α-tubulin. Statistical significance based on the mean normalized band intensity for each group (n = 3 independent samples) was determined by one-way analysis of variance, followed by Dunnett’s post-hoc analysis using Graphpad Prism 4.0. Differences were considered statistically significant when p<0.05.

## Results

### Principal Component Analysis

**Figure 1:**
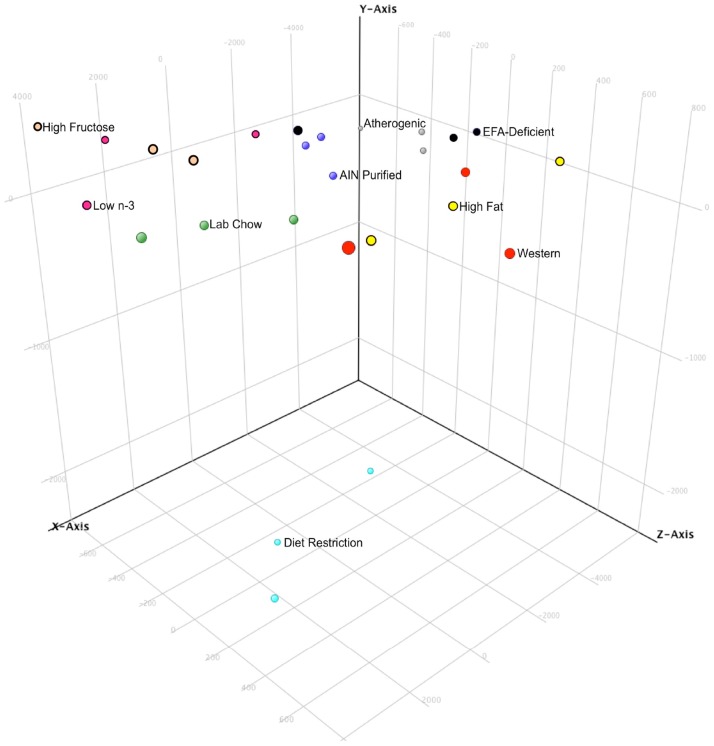
3D Scatter Plot of the Principal component analysis (PCA). Samples are displayed in respect to the first three components and are colored with respect to diet. PCA analysis allows visual identification of patterns in the data and highlight similarities and differences between diets. PCA was performed using GeneSpring and was based on conditions.

In order to visualize patterns in the data and highlight similarities and differences between diets, a principal component analysis (PCA) was performed using GeneSpring (Agilent Technologies) and conditions were based on the diet consumed. Data are displayed in a 3D-Scatter Plot view (**Figure 1**). Samples are displayed in respect to the first three components (each axis is a principal component), and are colored by the diet labels. Diet restriction clearly differed the most from the other diets. The biological replicates for each diet occupied similar 3D space indicating good consistency between replicates.

### Transcriptional Profiling of the Effect of Diet on Liver Gene Expression

**Figure 2:**
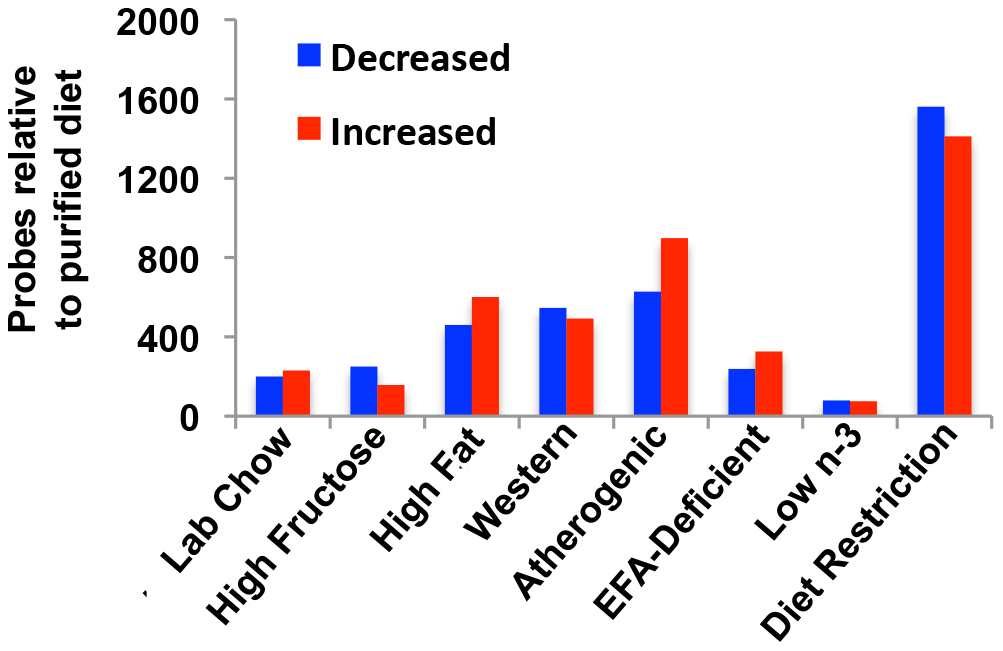
Number of probes that detected a significant change in gene expression by each diet compared to the AIN-93 purified control diet.

**Figure 3:**
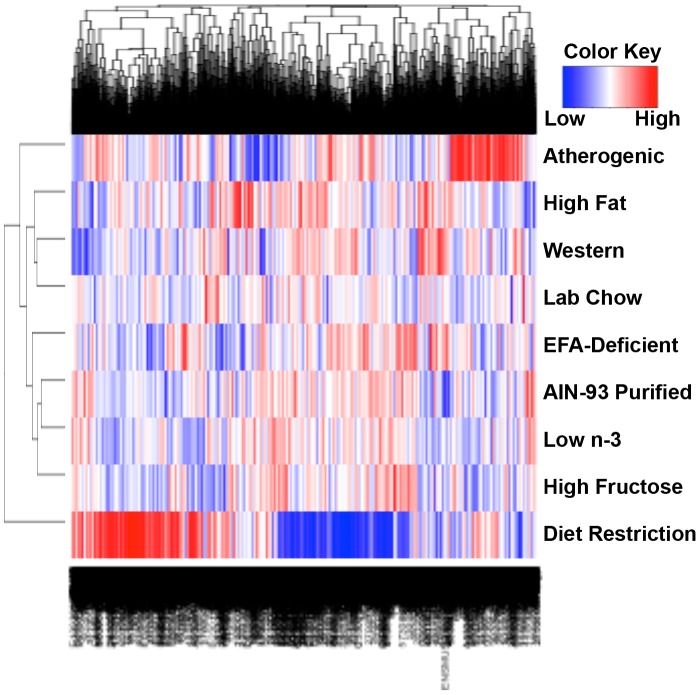
One-way hierarchical cluster map of the 3,734 genes that changed significantly by at least one diet compared to the AIN-93 purified control diet.

The effect of diet on gene transcription profiles in livers of mice was characterized by microarray. Each array contained 45,101 probes of which 20,449 correlated with unique gene symbols. There were 6,681 unique genes that were detected with a signal intensity higher than the threshold of log_2_(100) in at least one diet and were therefore selected for further analysis. When each diet was compared to the AIN-93 purified control diet using LIMMA, 3,734 unique genes were changed significantly by at least one of the diets. **Figure 2** illustrates the number of probes significantly increased or decreased by each diet compared to the AIN-93 purified control diet. One-way hierarchical clustering of the 3,734 genes that changed by at least one diet identified diet restriction to be the most dissimilar to the other diets, whereas the low n-3 diet was the most similar to the AIN-93 purified control diet (**Figure 3**). The atherogenic diet was the second most different diet from the purified control diet and did not cluster with any of the other diets. Surprisingly, the lab chow (natural ingredient diet) currently used in our animal facility was not similar to the AIN-93 purified control diet, but was most similar to the Western diet.

### Gene Set Enrichment Analysis

Gene set enrichment analysis was performed to define pathways that were affected by diet. Of interest, gene sets involved in glutathione metabolism, fatty acid metabolism, oxidative phosphorylation, immune responses, cholesterol biosynthesis, and ABC transporters were affected by many of the diets (**Table 2**) and hence became the focus of further analyses. The entirety of the GSEA results can be found in **Table S1**.

**Table 2:** Pathway analysis of genes significantly changed by at least one diet using GSEA software.

Diet	Pathways Increased	NES	FDR	Pathways Decreased	NES	FDR
**Lab Chow**	Glutathione Metabolism	2.57	0.000			
	Fatty Acid Metabolism	1.67	0.065			
**High Fructose**	Oxidative Phosphorylation	1.96	0.015	Complement & Coagulation Cascade	−1.18	0.081
	Fatty Acid Biosynthesis	1.42	0.150	Toll-like Receptor Signaling	−1.66	0.100
	Glutathione Metabolism	1.79	0.030			
	PPAR Signaling Pathway	1.43	0.147			
	Pahogenic E.coli Infection	1.65	0.070			
**High Fat**	ABC Transporters	1.83	0.008			
	Biosynthesis of Cholesterol	2.21	0.000			
	Fatty Acid Metabolism	2.51	0.000			
	Glutathione Metabolism	2.03	0.001			
	PPAR Signaling	2.19	0.000			
**Western**	Fatty Acid Metabolism	2.45	0.000	Biosynthesis of Cholesterol	−2.55	0.000
	ABC Transporters	2.26	0.000	Leukocyte Transendothelial Migration	−1.70	0.117
	Glutathione Metabolism	1.70	0.035			
	PPAR Signaling Pathway	2.09	0.001			
**Atherogenic**	ABC Transporters	1.93	0.002	Biosynthesis of Cholesterol	−2.74	0.000
	Glutathione Metabolism	1.96	0.002	Oxidative Phosphorylation	−1.62	0.143
	Antigen Processing & Presentation	2.04	0.003			
**EFA-deficient**	Biosynthesis of Cholesterol	1.97	0.014	Complement & Coagulation Cascade	−1.94	0.013
	Fatty Acid Biosynthesis	1.55	0.171			
	PPAR Signaling Pathway	1.58	0.157			
	Pathogenic E.coli Infection	1.65	0.127			
**Low n-3**	Oxidative Phosphorylation	1.95	0.017	None with FDR <25%		
	Glutathione Metabolism	1.77	0.066			
	Pathogenic E.coli Infection	1.49	0.188			
**Diet Restriction**	ABC Transporters	1.78	0.039	Biosynthesis of Cholesterol	−1.48	0.242
	Oxidative Phosphorylation	1.55	0.178	Leukocyte Transendothelial Migration	−1.43	0.249
	Glutathione Metabolism	2.31	0.000	Pathogenic E.coli Infection	−1.42	0.247

NES: normalized enrichment score (analyzed using KEGG pathway gene sets in the GSEA software); FDR: false discovery rate (only gene sets with FDR <25% are shown).

### The Effect of Diet on Lipid Homeostasis

**Figure 4:**
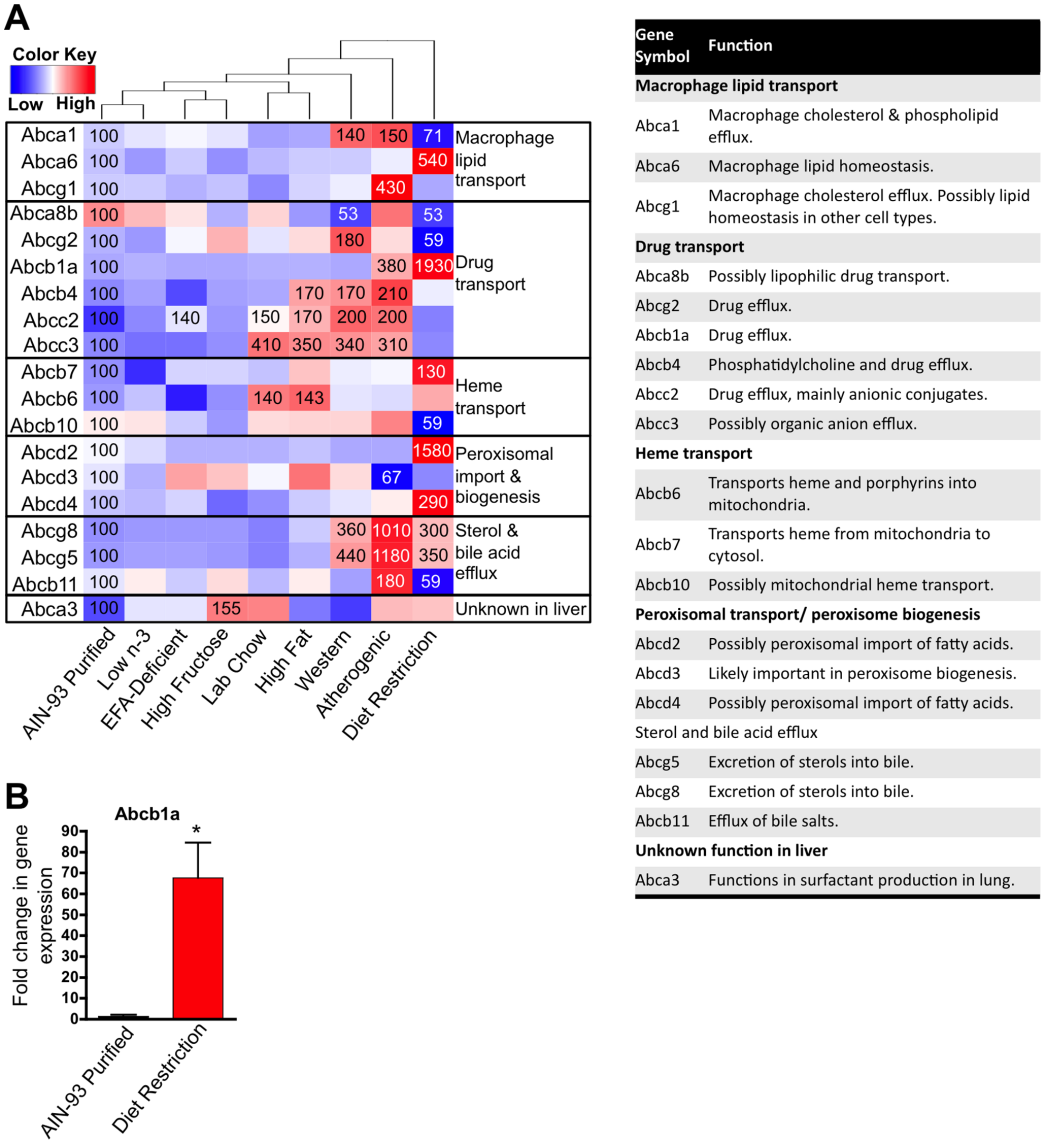
Effect of diet on the expression of ABC transporter genes. **A** One-way hierarchical cluster map of ABC transporter genes that changed significantly by at least one diet. mRNA expression of genes that were statistically different from those in mice fed the AIN-93 purified control diet have the percent indicated within the heatmap square (control = 100%). **B** RT-qPCR results validating the microarray result of Abcb1a. Data statistically significant from control are denoted with an asterisk (*).

ABC transporters transport a wide variety of substrates including lipids and sterols across extra- and intracellular membranes. Several ABC transporters play a key role in maintaining lipid homeostasis; therefore, it is not surprising that the expression of many ABC transporters was sensitive to diet. Of the 50 mouse ABC transporters examined, 26 were above the threshold of detection in mouse liver, and the expression of 19 were significantly changed by at least one diet and are presented in **Figure 4**A. Surprisingly, diet restriction substantially upregulated Abcb1a (Mdr1a/P-gp) to 1,930% of control. This effect was confirmed by qPCR (**Figure 4B**).

**Figure 5:**
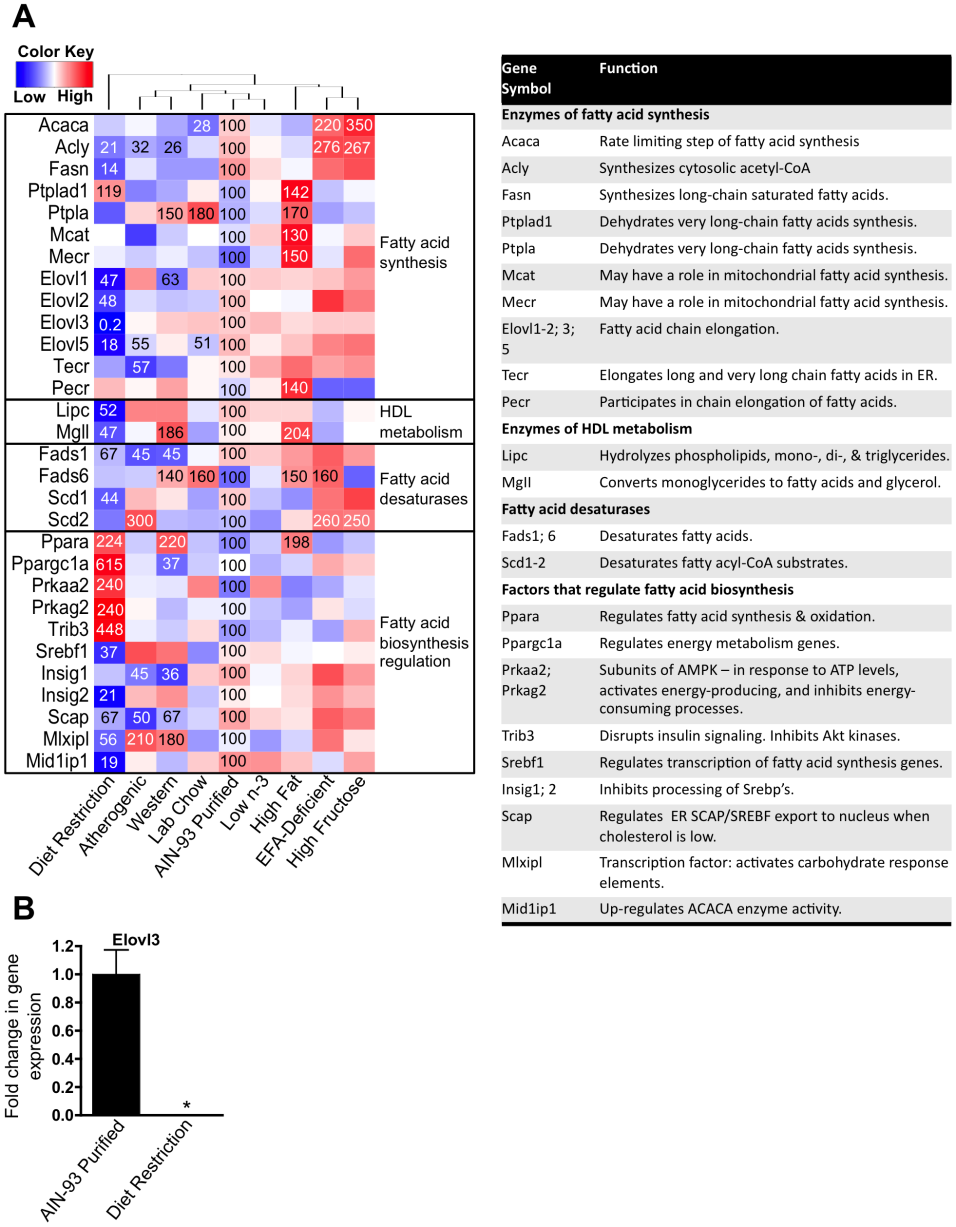
Effect of diet on the expression of fatty acid biosynthesis genes. **A** One-way hierarchical cluster map of fatty acid biosynthesis genes that changed significantly by at least one diet. mRNA expression of genes that were statistically different from those in mice fed the AIN-93 purified control diet have the percent indicated within the heatmap square (control = 100%). **B** RT-qPCR results validating the microarray result of Elovl3. Data statistically significant from control are denoted with an asterisk (*).

The effect of diet on fatty acid metabolic processes was determined using the gene ontology gene set GO:0006633 (Fatty acid biosynthetic process, 308 genes), but excluding genes exclusively involved in leukotriene biosynthesis, wax biosynthesis, prostaglandin biosynthetic process, and the cyclooxygenase pathway (resulting list = 176 genes). From this list, 30 genes for fatty acid synthesis (**Figure 5**), 18 genes for mitochondrial β-oxidation (**Figure S1**), and 12 genes for peroxisomal β-oxidation (discussed below) were significantly changed by at least one diet.

The rate-limiting step of fatty acid synthesis is the enzyme acetyl-CoA carboxylase (Acaca). Abundance of the mRNA for this enzyme was only increased by the high-fructose (350% of control) and the EFA-deficient (220% of control) diets, but decreased (28% of control) by the lab chow diet (**Table S2** and **Figure 5**). Hierarchical clustering of fatty acid synthesis genes indicated that the high-fructose and EFA-deficient diets affected the expression of similar genes. Interestingly, diet restriction affected fatty acid synthesis genes the most, including Elovl3 (0.2% of control) – a gene positively associated with diet-induced obesity (**Figure 5**). This result was confirmed by qPCR (**Figure 5**).

Mitochondrial β-oxidation is the process that breaks down acyl-CoA molecules into acetyl-CoA units for use in the citric acid cycle, a way for the cell to mobilize energy from fatty acids. The rate-limiting step of this process is carnitine acyltransferase (Cpt1a), which was increased only by the high-fat diet (210% of control) (**Table S2** and **Figure S1**). The high-fat and Western diets affected the most number of mitochondrial β-oxidation genes. Interestingly, the high-fat diet, Western diet, and diet restriction clustered together in the one-way hierarchical clustering of mitochondrial β-oxidation genes (**Figure S1**).

**Figure 6:**
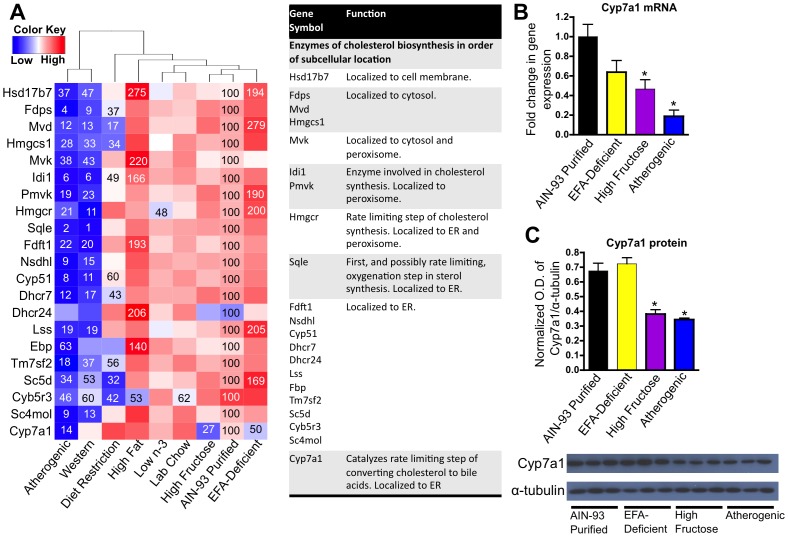
Effect of diet on the expression of cholesterol biosynthesis genes. **A** One-way hierarchical cluster map of cholesterol biosynthesis genes that changed significantly by at least one diet. mRNA expression of genes that were statistically different from those in mice fed the AIN-93 purified control diet have the percent indicated within the heatmap square (control = 100%). **B** RT-qPCR results validating the microarray result of Cyp7a1. Data statistically significant from control are denoted with an asterisk (*). **C** Western blot analysis of Cyp7a1 protein expression validating our mRNA findings. Top panel is a bar graph of the optical densities measured by ImageJ; bottom panel is representative western blots of Cyp7a1 and α-tubulin.

Cholesterol biosynthesis is directly regulated by circulating levels of cholesterol through Srebp-Scap-Insig1 signaling. Expression of the enzyme responsible for the rate limiting step of cholesterol biosynthesis, HMG-CoA reductase (Hmgcr), was significantly decreased by feeding the atherogenic diet (21% of control), the Western diet (11% of control), and the low n-3 diet (48% of control) (**Table S2** and **Figure 6**). Using the gene ontology gene set GO:0006695 Cholesterol biosynthetic process, 21 out of 37 genes tested were significantly changed by at least one diet. One-way hierarchical clustering of these 21 genes (**Figure 6**) shows that the atherogenic diet clustered closely with the Western diet. Both diets resulted in a decrease in the mRNA of a majority of these cholesterol synthesis genes. Intriguingly, not only the atherogenic diet, but the high-fructose and EFA-deficient diets also decreased Cyp7a1, the rate limiting step of cholesterol metabolism into bile acids, to 14%, 27%, and 50% of control, respectively. The decrease in mRNA and protein of Cyp7a1 by the high fructose and atherogenic diets was confirmed by qPCR (Figure 6B) and western blot analysis (Figure 6C), respectively.

**Figure 7:**
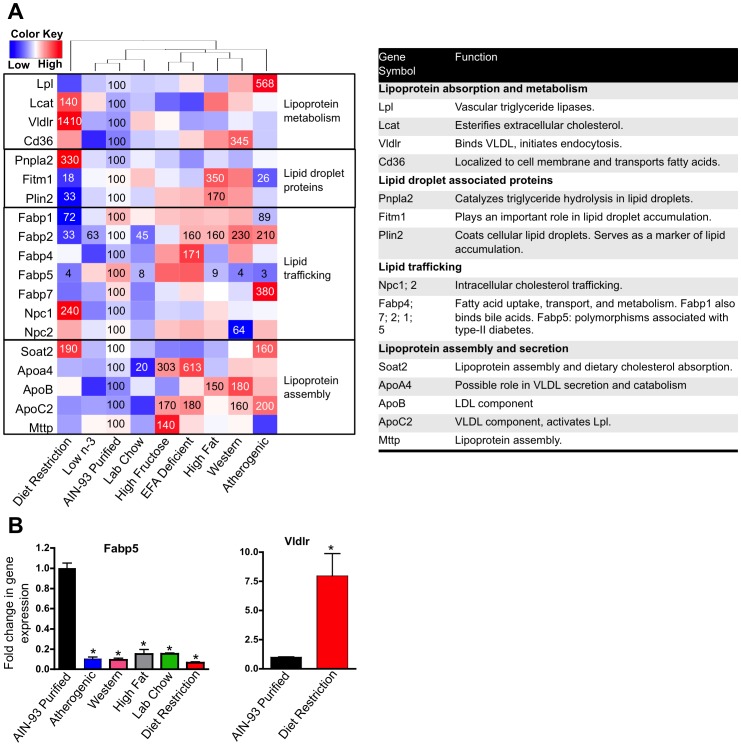
Effect of diet on the expression of genes involved in lipid trafficking, VLDL secretion, and lipid droplet formation. **A** One-way hierarchical cluster map of genes involved in lipid trafficking, VLDL secretion, and lipid droplet formation that changed significantly by at least one diet. mRNA expression of genes that were statistically different from those in mice fed the AIN-93 purified control diet have the percent indicated within the heatmap square (control = 100%). B RT-qPCR results validating the microarray results of Fabp5 and Vldlr. Data statistically significant from control are denoted with an asterisk (*).

A list of genes involved in lipid trafficking, lipid droplet formation and storage, VLDL secretion, and lipoprotein/chylomicron remnant uptake into hepatocytes was compiled by literature searches of genes implicated in these processes. Out of 40 genes selected for analysis, the mRNA of 19 were significantly changed by at least one diet compared to the AIN-93 purified control diet. Diet restriction affected the expression of the most number of genes and the magnitude of gene expression changes more than the other diets did within this gene set (**Figure 7**). Fatty acid binding proteins (FABPs) are most active in long-chain fatty acid uptake and metabolism (liver, intestine), oxidation (kidney, heart, skeletal muscle), and storage (adipose) [26]. Fabp5 is expressed in many tissues including the liver [27] and studies in mice null for both Fabp4 and Fabp5 demonstrate a role for Fabp5 in systemic glucose and lipid homeostasis as these mice are substantially protected from the metabolic syndrome [28]. Additionally, polymorphisms in human FABP5 are associated with type 2 diabetes [29]. Interestingly, Fabp5 changed markedly across diets as it was decreased by the Western (4% of control), atherogenic (3% of control), high-fat (9% of control), lab chow (8% of control), and diet restriction (4% of control). Under basal conditions, the VLDL receptor, Vldlr, is expressed at very low levels in liver [30]; however is induced in liver during ER stress, which is thought to contribute to hepatic steatosis [31]. Surprisingly, only diet restriction increased Vldlr expression in liver (1,410% of control).

### The Effect of Diet on Pathways Involved in Cellular Oxidative Stress

**Figure 8:**
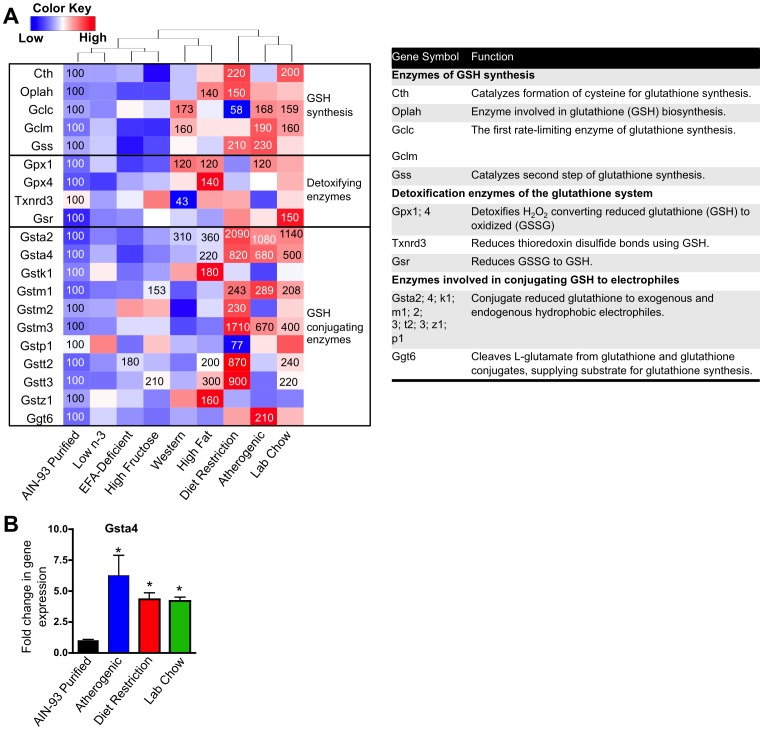
Effect of diet on the expression of glutathione metabolism genes. **A** One-way hierarchical cluster map of glutathione metabolism genes that changed significantly by at least one diet. mRNA expression of genes that were statistically different from those in mice fed the AIN-93 purified control diet have the percent indicated within the heatmap square (control = 100%). **B** RT-qPCR results validating the microarray result of Gsta4. Data statistically significant from control are denoted with an asterisk (*).

An increase in cellular oxidative or electrophilic stress induces expression of genes in glutathione synthesis, glutathione peroxidase, and glutathione conjugation [32], [33]. According to our GSEA results, all diets increased the expression of glutathione metabolism genes compared to the AIN-93 purified control diet, except the EFA-deficient diet (**Table 2**). However, the rate-limiting step of glutathione metabolism, glutamate-cysteine ligase (Gclc), was only increased by the Western (173% of control), atherogenic (168% of control), and lab chow (159% of control) diets (**Table S2** and **Figure 8**). The KEGG glutathione metabolism gene list was used to evaluate individual genes of glutathione metabolism. Of the 54 glutathione genes examined, the mRNA of 20 genes changed significantly by at least one diet, and are plotted in a one-way hierarchically clustered heatmap (**Figure 8**). The mRNA of the oxidative stress marker, Gsta4 [34], was increased by the atherogenic diet (680% of control), lab chow diet (500% of control), diet restriction (820% of control), and high-fat diet (220% of control). Together, these data indicate that diet restriction, atherogenic, Western, high-fat, and the lab chow diet possibly increase cell oxidant status compared to the AIN-93 purified control diet.

Physiological sources of cellular oxidants include the leakage of activated oxygen during oxidative phosphorylation, as well as the formation of H_2_O_2_ during peroxisomal β-oxidation. To gain insight into how these diets possibly increase the production of oxidative species in liver, one-way hierarchical clusterings of oxidative phosphorylation (**Figure S2**) and peroxisomal β-oxidation (**Figure S3**) genes were performed. Evaluation of 148 KEGG oxidative phosphorylation pathway genes provided 38 genes whose expression was significantly altered by at least one diet. A heatmap of these 38 genes is presented in **Figure S2**. Compared to the AIN-93 purified control diet, all of these mRNA expression changes were relatively small in magnitude, only differing by 63–140%. Next, we evaluated the effect of diet on genes involved in peroxisomal β-oxidation (**Figure S3**). The rate-limiting step of peroxisomal β-oxidation is acyl-CoA oxidase-1 (Acox1) and its mRNA was increased by the high-fat (181% of control) and Western (241% of control) diets (**Table S2** and **Figure S3**).

### The Effect of Diet on Inflammation

**Figure 9:**
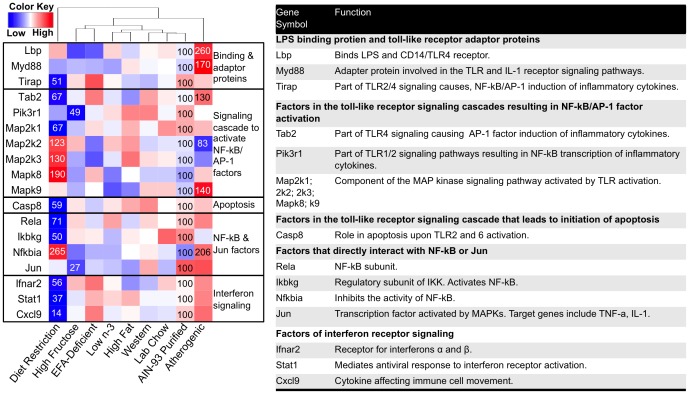
One-way hierarchical cluster map of toll-like receptor signaling genes that changed significantly by at least one diet. mRNA expression of genes that were statistically different from those in mice fed the AIN-93 purified control diet have the percent indicated within the heatmap square (control = 100%).

To evaluate the effect of diet on expression of inflammation genes, we evaluated gene lists from the KEGG including: toll-like receptor signaling (101 genes); complement and coagulation cascade (69 genes); and antigen processing and presentation (81 genes)+leukocyte transendothelial migration (115 genes). Of these 366 genes, the mRNA of 77 changed significantly by at least one diet and these data were used to generate 3 one-way hierarchical cluster maps (**Figure 9** **,** **10** **, and** **11**). The mRNA of 18 genes involved in toll-like receptor signaling were significantly changed by at least one diet with most of these genes being affected by diet restriction and the atherogenic diet (**Figure 9**). Interestingly, the atherogenic diet increased mRNA of the LPS binding protein, Lbp (260% of control) and a crucial adaptor protein needed for TLR signaling, Myd88 (170% of control). Overall, these mRNA changes suggest that toll-like receptor signaling pathways are up-regulated by an atherogenic diet.

**Figure 10:**
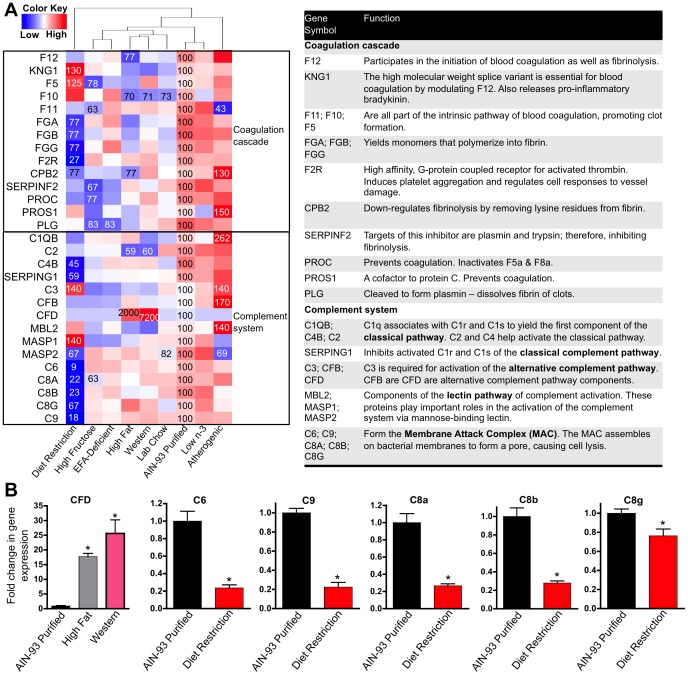
Effect of diet on the expression of complement and coagulation cascade genes. **A** One-way hierarchical cluster map of complement and coagulation cascade genes that changed significantly by at least one diet. mRNA expression of genes that were statistically different from those in mice fed the AIN-93 purified control diet have the percent indicated within the heatmap square (control = 100%). **B** RT-qPCR results validating the microarray results of CFD, C6, C9, C8a, C8b, and C8g. Data statistically significant from control are denoted with an asterisk (*).

**Figure 11:**
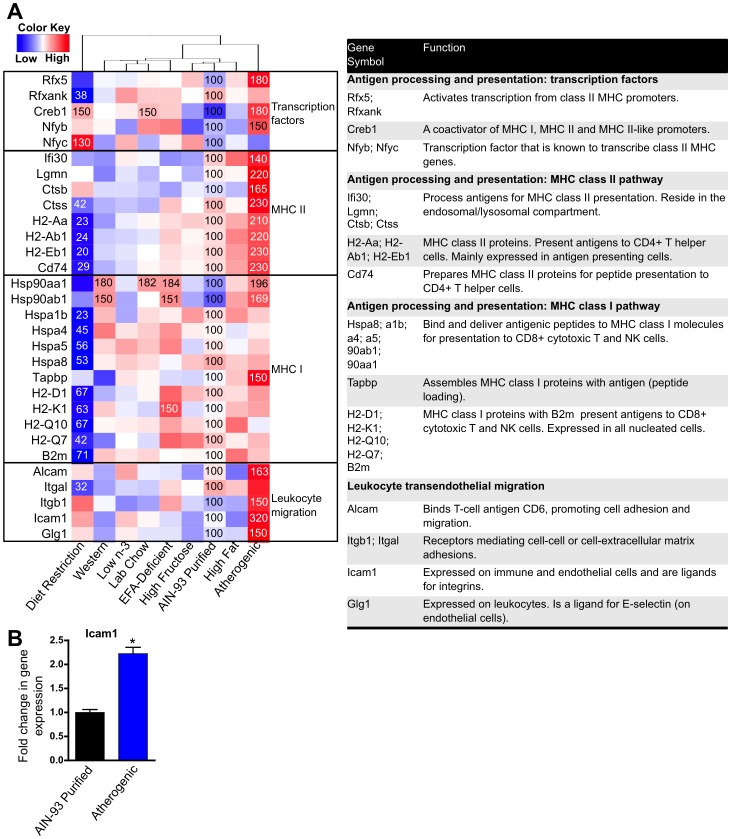
Effect of diet on the expression of antigen processing and presentation, and leukocyte transendothelial migration genes. **A** One-way hierarchical cluster map of antigen processing and presentation and leukocyte transendothelial migration genes that changed significantly by at least one diet. mRNA expression of genes that were statistically different from those in mice fed the AIN-93 purified control diet have the percent indicated within the heatmap square (control = 100%). **B** RT-qPCR results validating the microarray result of Icam1. Data statistically significant from control are denoted with an asterisk (*).

The mRNA of 29 genes of the KEGG complement and coagulation cascade gene list were significantly changed by at least one diet, with diet restriction affecting the most number of these genes (**Figure 10**). Diet restriction had the largest effect on the expression of complement genes involved in the formation of the membrane attack complex (forms a pore on bacterial membranes) including C6 (9% of control), C9 (18% of control), C8A (22% of control), C8B (23% of control), and C8G (67% of control). Interestingly, the Western diet and high-fat diet markedly induced the alternate complement pathway component CFD by 7,200% and 2,000%, respectively. Overall, these mRNA expression changes suggest that pathways of the complement system are down-regulated by diet restriction, but up-regulated by the high fat diets.

Changes in dietary composition affected the expression of 25 KEGG antigen processing and presentation genes and 5 genes involved in leukocyte transendothelial migration (**Figure 11**). Four of the five leukocyte transendothelial migration genes had increased mRNA expression by the atherogenic diet including the following genes that all promote leukocyte binding to endothelial cells: Icam1 (320% of control, known marker of inflammation), Alcam (163% of control), Glg1 (150% of control), and Itgb1 (150% of control). These data suggest that an atherogenic diet may promote leukocyte transendothelial migration.

### Relationships between Diets

**Figure 12:**
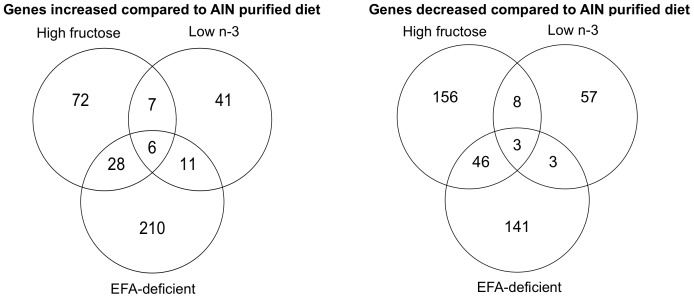
Venn diagrams of genes increased or decreased in livers of mice fed a high-fructose, low n-3, or EFA-deficient diet.

Throughout the pathways evaluated in this study, the high-fructose diet and the EFA-deficient diet often clustered together having similar effects on gene expression. These observations were interesting as the EFA-deficient diet was also similar in composition to the low n-3 diet. To determine the degree of similarity between these diets, Venn diagrams were created (**Figures 12**) to analyze all 3,734 genes that changed significantly by at least one diet. Comparing the high-fructose, EFA-deficient and low n-3 diets, for both increased and decreased genes, the EFA-deficient diet had more in common with the high-fructose diet than the low n-3 diet. (**Figure 12**). These data suggest that the driving force regulating gene expression by these diets is most likely sugar content, not deficiency in essential fatty acids.

## Discussion

The liver plays an important role in maintaining nutrient homeostasis by buffering the daily flux of sustenance between meals. However, it is believed that chronic consumption of a Western-type diet (high in sugar, fat, and overall calories) can disrupt the liver’s ability to maintain nutrient homeostasis, thereby leading to steatosis and the metabolic syndrome. Our understanding of the causal relationship between diet and hepatic metabolic homeostasis is limited. In order to gain a better understanding of the early effects of diet on hepatic molecular pathways involved in diet-induced liver dysfunction, we compared the effects of various diets on overall hepatic gene expression.

Diet restriction and the atherogenic diet were associated with very different gene expression patterns compared to the other diets evaluated in this study, and to each other. Of the 9 diets assessed, diet restriction is the only one known to alleviate fat accumulation in the liver [35]. This may explain why diet restriction was associated with markedly different hepatic gene expression patterns in comparison to the other evaluated diets. It was interesting that the atherogenic diet caused changes in gene expression that were quite different from those associated with a Western diet. These two diets were very similar in macronutrient content, except for the inclusion of CA and a much higher cholesterol concentration in the atherogenic diet. The inclusion of CA in an experimental atherogenic diet is necessary to produce atherosclerosis, but also exacerbates fatty liver development and suppresses bile acid synthetic genes, resulting in cholesterol accumulation in the liver [36], [37], [38]. Therefore, the inclusion of CA may explain why the atherogenic diet caused marked changes to hepatic gene expression in comparison to the other diets evaluated in this study.

The present data demonstrate for the first time that mice on a high fructose-containing diet have decreased hepatic Cyp7a1 mRNA and protein (rate limiting step of cholesterol metabolism into bile acids). A recent study showed that a single oral gavage of glucose to fasted mice markedly induced the mRNA of Cyp7a1 in liver [39], which is likely a normal, nutrient-driven response. Our data suggest that chronic, high-fructose consumption alters this normal response, leading to increased hepatic cholesterol accumulation. Cholesterol metabolism may play a role in the development of NAFLD. For example, in steatotic livers, cholesterol biosynthesis is still activated despite a cholesterol overload in hepatocytes, indicating that cholesterol metabolism is dysregulated [40], [41]. Additionally, in mice, dietary cholesterol exacerbates hepatic steatosis [42]. Therefore, suppression of Cyp7a1 leading to hepatic cholesterol accumulation may be a novel mechanism of high-fructose diet-induced fatty liver.

Diet restriction was associated with decreased expression of a number of fatty acid synthesis genes. Of interest was the robust down-regulation of Elovl3. Elovl3 is implicated in diet-induced obesity [43]. Ablation of Elovl3 in mice causes resistance to diet-induced obesity, and reduces hepatic lipogenic gene expression and triglyceride content. The results from the present study support a role for Elovl3 in diet-induced obesity.

ABC transporters are a family of proteins known to regulate lipid transport. One of our study’s novel findings is the increased expression of Abcb1a (Mdr1a; P-gp) in both the atherogenic diet and diet restriction. The function of Abcb1a in liver cells is to efflux drugs and other xenobiotics into bile; although an endogenous function for this transporter is not known. Abcb1a may have a role in cholesterol metabolism, because Abcb1a/b null mice have elevated fecal cholesterol concentrations [44]. Abcb1a/b may act as a cholesterol “dock” which facilitates the transfer of cholesterol from canalicular bile back into hepatocytes, counterbalancing Abcg5/8-mediated cholesterol efflux [45]. Thus, Abcb1a/b-mediated cholesterol uptake from bile may act to prevent excess loss of cholesterol. The present observation that diet restriction induces Abcb1a is supportive of this hypothesis. However, Abcb1a mRNA was also increased in the atherogenic diet. In this instance, elevated Abcb1a may contribute to pathological cholesterol accumulation in the liver.

An important finding from our study was that the composition of the animal diet, including the use of natural versus purified ingredients, can markedly affect hepatic gene expression. An example of this is Fabp5, which was decreased in high-fat containing diets, diet restriction, and the natural ingredient diet (Lab Chow). These results contradict the findings of another study, which found that mice fed a Western diet caused increased hepatic Fabp5 expression [46]. One possible explanation for the discrepancy between these two studies is the use of natural versus purified ingredients. The control diet used by Hoekstra *et al.* (2006) was made from all natural ingredients, however the Western-type diet was made from purified ingredients. Our study showed a large decrease in Fabp5 expression by the natural ingredient diet (Lab chow) compared to the AIN-93 purified ingredient diet. Therefore, it is plausible that the increase in Fabp5 expression observed in the Hoekstra *et al.* (2006) study is due to comparing a purified diet to a natural ingredient diet. Thus, our study emphasizes diet composition as an important consideration in the design of experiments using laboratory animals.

Diet restriction caused decreased expression of genes involved in the formation of lipid droplets, and increased expression of genes involved in the dissolution of lipid droplets. Lipid droplets serve as a reservoir of fatty acids and cholesterol esters, which are hydrolyzed by cytosolic lipases when the cellular need arises. Pnpla2 is a triglyceride lipase responsible for hydrolyzing triglycerides from lipid droplets providing free fatty acids and glycerol for cellular use; additionally, mutations in this gene lead to triglyceride accumulation in tissues, including the liver [47]. Interestingly, the present data are the first to show that diet restriction increased expression of Pnpla2– a possible, novel mechanism behind the beneficial effects of diet restriction in alleviating fatty liver. Diet restriction also increased expression of the VLDL receptor (Vldlr), which is surprising as the hepatic induction of this gene during ER stress is associated with the development of steatosis in mice [31]. In severely obese humans, rapid weight loss induced by jejuno-ileal bypass surgery or severe calorie restriction can result in increased fatty infiltration in liver [48]. The present observation that diet restriction can induce Vldlr expression may explain why severe calorie restriction in overweight individuals can worsen fatty liver.

There is a strong association between severity of NASH and degree of oxidative stress [reviewed in: [49]]. The major defense against oxidative stress in the liver is glutathione [reviewed in: [50]]. Both diet restriction and the atherogenic diet increased genes involved in the synthesis of glutathione (GSH) and glutathione-S transferase (Gst). In particular, Gsta2, which is induced during oxidative stress [51], was substantially induced by high-fat diets as well as diet restriction. Although oxidative stress was not measured directly, these results suggest that oxidative stress is present in livers of mice fed the high fat diets and following diet restriction. Our data also suggest that the high fat and Western diets may lead to cellular accumulation of ROS in part by increasing peroxisomal β-oxidation, as a number of genes in this pathway were induced. It was surprising that diet restriction also increased oxidative stress pathways, lending a paradox to the question of whether increased oxidative stress is a causative factor in the pathogenesis of NAFLD. Early calorie restriction causes increased mobilization of lipids from peripheral fat stores to the liver for energy production, simulating a high fat diet. Therefore, it is possible that diet restriction and the high fat diets could cause similar changes in expression of genes involved in oxidative stress response. To the best of our knowledge, this is the first report to identify induction of Gst genes in response to changes in multiple dietary macronutrients, suggesting that Gst enzymes play a major role in maintaining redox balance in the liver following nutrient uptake.

Dietary restriction is associated with extended life span in multiple species, however the mechanisms remain unknown. An emerging notion is the redox-stress hypothesis, which proposes that age-related diseases are caused by a progressive cellular accumulation of pro-oxidants, leading to the consequent disruption of redox-regulated signaling mechanisms (reviewed in [52]. Supporting this hypothesis, many studies report diet restriction to have little effect on mitochondrial ROS production and antioxidative enzyme activity; but an increase in glutathione levels (reviewed in [53]. Although the duration of dietary restriction used in our study is moderate compared to longevity studies, our data lends support to the redox-stress hypothesis of aging because diet restriction increased transcript abundance of genes involved in the synthesis of glutathione.

The hallmark of NAFLD progression to NASH is inflammation. Many proinflammatory genes were induced by the atherogenic diet, which is consistent with other reports [54], [55]. It is known that atherogenic diet-induced hepatic inflammation is partially dependent on toll-like receptor 4 (Tlr4) activation [54]. Tlr4 is a cell surface receptor that detects lipopolysaccharide from gram-negative bacteria, and induces signaling cascades that ultimately induce cytokine production as a component of an inflammatory response. Our study adds additional evidence that an atherogenic diet may elicit a Tlr4 response, as we observed increased levels of LPS binding protein (Lbp) and Myd88, which are both involved in toll-like receptor signaling. It is unknown whether the ligand that activates Tlr4 under atherogenic feeding is an intestinal bacterial translocation product or a component of the diet itself. Further experiments with germ-free mice would help clarify this question.

Our data demonstrate for the first time that diet restriction decreases a number of complement factor genes. The complement system is part of the innate immune response, and act by inducing chemotaxis of inflammatory cells and enhancing their phagocytic capacity. Complement factors are primarily synthesized in the liver. The fact that diet restriction suppressed the expression of several complement factors suggests an overall lower level of inflammation in livers of diet-restricted mice. In line with this, calorie restriction alleviates hepatic inflammation in a mouse model of diet-induced obesity [56], and suppresses auto-antibody formation in a model of immune nephritis [57], [58]. Clearly, diet restriction seems to be protective in inflammation-related pathologies.

It should be noted that, although relative mRNA levels obtained via microarray comparisons are an invaluable resource, discretion must be used when inferring changes at protein levels. Additionally, when animals are fasted or fed, multiple factors impinge upon the metabolic program of the liver. Hormonal changes should also be considered in the disposition of intermediary metabolism, regulating at the levels of both catalytic efficiency and gene expression [59], [60]. Therefore, because feeding conditions affect these hormonal changes, it is important to note that mice in this study were in a “fed” state when tissues were collected.

Currently, there is no effective treatment for steatosis. Lifestyle modifications remain the best therapeutic option. To develop appropriate treatments, knowledge of the cellular and molecular mechanisms leading to hepatic steatosis is essential. By providing a large database of gene expression changes brought about by various diets, our study has provided new insights into steatosis progression and homeostatic regulation of nutrient metabolism. A better understanding of the biochemical and pathological changes that cause diet-induced NAFLD may lead to novel therapies to better manage or treat obesity-associated health conditions.

## Supporting Information

Figure S1 **One-way hierarchical cluster map of mitochondrial β-oxidation genes that changed significantly by at least one diet.** mRNA expression of genes that were statistically different from those in mice fed the AIN-93 purified control diet have the percent indicated within the heatmap square (control = 100%).
(PDF)

Figure S2 **One-way hierarchical cluster map of oxidative phosphorylation enzymes that changed significantly by at least one diet.** mRNA expression of genes that were statistically different from those in mice fed the AIN-93 purified control diet have the percent indicated within the heatmap square (control = 100%).
(PDF)

Figure S3 **One-way hierarchical cluster map of peroxisomal β-oxidation genes that changed significantly by at least one diet.** mRNA expression of genes that were statistically different from those in mice fed the AIN-93 purified control diet have the percent indicated within the heatmap square (control = 100%).
(PDF)

Table S1 **Gene set enrichment analysis (GSEA). List of all functional gene sets correlated with diet that have an FDR<0.25.**
(PDF)

Table S2 **The effect of diet on the rate limiting step or biomarker of key pathways.**
(PDF)

Table S3 **Oligonucleotide primers used for qPCR.**
(PDF)
