# Supplementary material for: Effect of Diet on Expression of Genes Involved in Lipid Metabolism, Oxidative Stress, and Inflammation in Mouse Liver–Insights into Mechanisms of Hepatic Steatosis
Source: PLoS One. 2014 Feb 14;9(2):e88584. doi: 10.1371/journal.pone.0088584 (PMC3925138; doi:10.1371/journal.pone.0088584)
Supplement: Figure S1 — One-way hierarchical cluster map of mitochondrial β-oxidation genes that changed significantly by at least one diet. mRNA expression of genes that were statistically different from those in mice fed the AIN-93 purified control diet have the percent indicated within the heatmap square (control = 100%). (PDF) [file pone.0088584.s001.pdf]

Figure S1

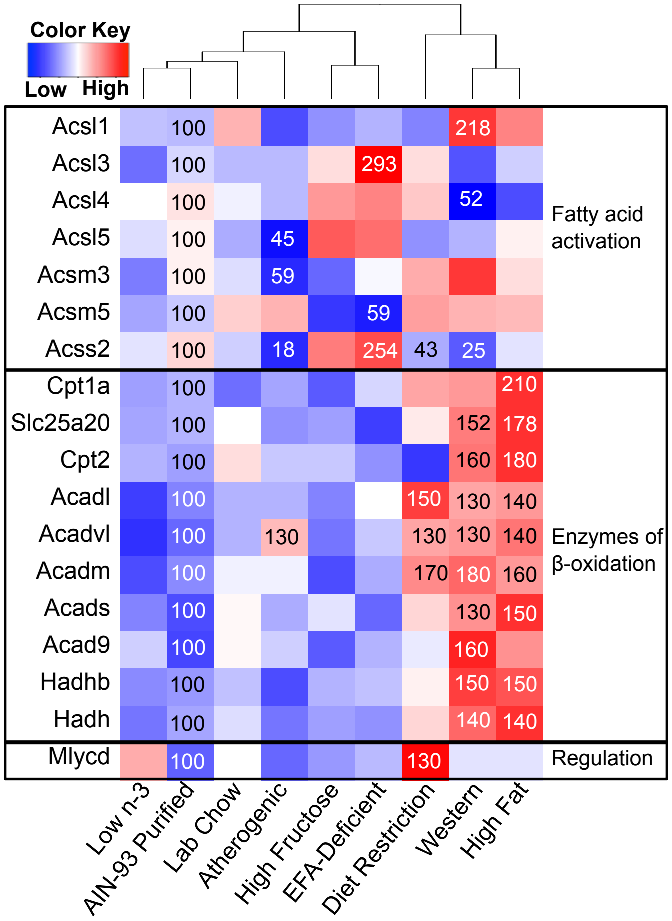

Gene Symbol	Function
**Enzymes that activate fatty acids for β-oxidation**	
Acsl1; 3; 4; 5	Convert long-chain fatty acids to acyl-CoA esters. Localized to mitochondria and peroxisome.
Acsm3; 5	Catalyzes addition of CoA onto medium chain fatty acids for mitochondrial import.
Acss2	Activates acetate. Localized to cytosol.
**Enzymes of mitochondrial β-oxidation**	
Cpt1a	The key enzyme in the carnitine-dependent transport of fatty acids into mitochondria.
Slc25a20	Transports acyl-carnitines into mitochondrial matrix for β-oxidation.
Cpt2	Catalyzes the removal of carnitine from acyl-CoAs in the mitochondrial matrix.
Acadl; m; vl; s; 9	Catalyze the initial steps of mitochondrial β-oxidation of long medium, very long, and short chain fatty acids.
Hadhb	Enzyme of mitochondrial β-oxidation of long chain fatty acids.
Hadh	Enzyme of mitochondrial β-oxidation of short chain fatty acids.
**Factors that regulate rate of β-oxidation**	
Mlycd	Catabolizes malonyl-CoA. Malonyl-CoA inhibits mitochondrial fatty acid transport.

Figure S1. One-way hierarchical cluster map of mitochondrial β-oxidation that changed significantly by at least one diet. mRNA expression of genes that were statistically different from those in mice fed the AIN-93 purified control diet have the percent indicated within the heatmap square (control = 100%).
