# Supplementary material for: Effect of Diet on Expression of Genes Involved in Lipid Metabolism, Oxidative Stress, and Inflammation in Mouse Liver–Insights into Mechanisms of Hepatic Steatosis
Source: PLoS One. 2014 Feb 14;9(2):e88584. doi: 10.1371/journal.pone.0088584 (PMC3925138; doi:10.1371/journal.pone.0088584)
Supplement: Figure S2 — One-way hierarchical cluster map of oxidative phosphorylation enzymes that changed significantly by at least one diet. mRNA expression of genes that were statistically different from those in mice fed the AIN-93 purified control diet have the percent indicated within the heatmap square (control = 100%). (PDF) [file pone.0088584.s002.pdf]

Figure S2

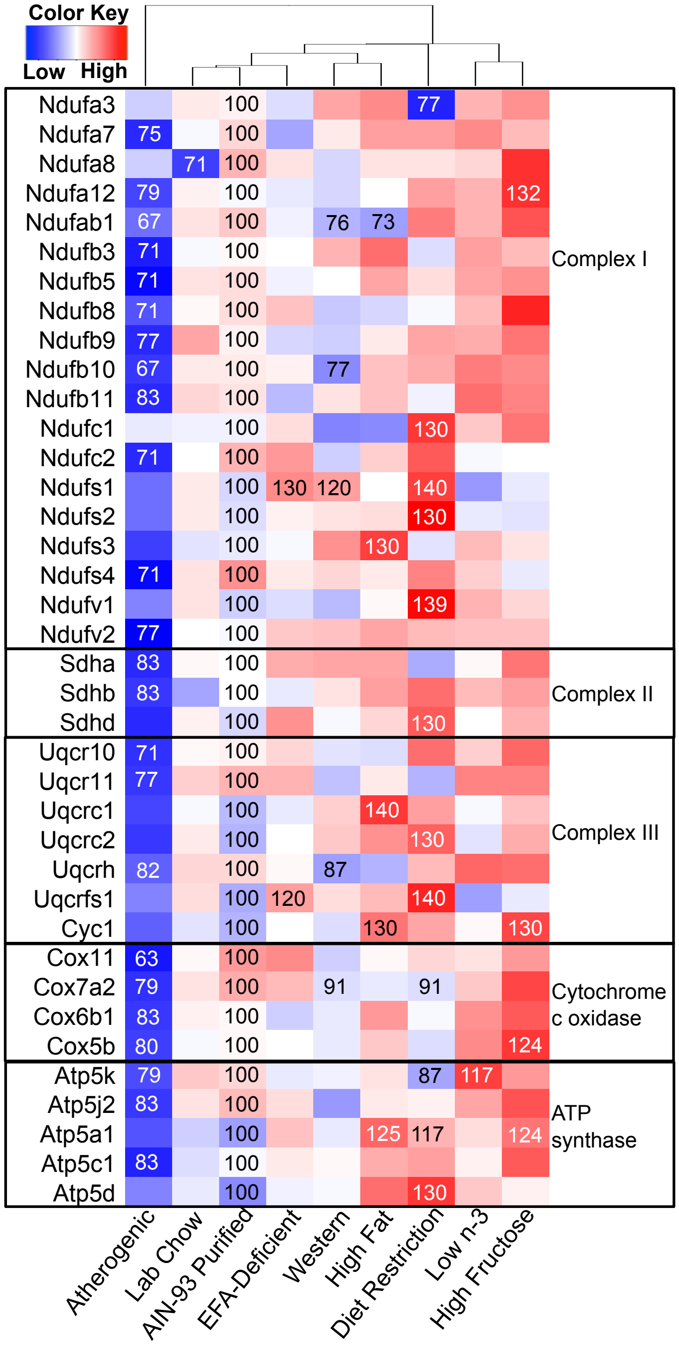

Gene Symbol	Function
**Nuclear encoded subunits of NADH dehydrogenase (Complex I)**	
Ndufa3<br>Ndufa7<br>Ndufa8<br>Ndufa12<br>Ndufab1<br>Ndufb3<br>Ndufb5<br>Ndufb8<br>Ndufb9<br>Ndufb10<br>Ndufb11<br>Ndufc1<br>Ndufc2<br>Ndufs1<br>Ndufs2<br>Ndufs3<br>Ndufs4<br>Ndufv1<br>Ndufv2	Complex I functions in the transfer of e^- from NADH to the respiratory chain. This complex translocates protons across the inner mitochondrial membrane to help build the electrochemical potential used to produce ATP. This complex consists of subunits encoded by nuclear genes and mitochndrial genes. Complex I is also a source of reactive oxygen species (superoxide and hydrogen peroxide).
**Subunits of succinate dehydrogenase complex (complex II)**	
Sdha<br>Sdhb<br>Sdhd	Composed of 4 nuclear encoded subunits. Transfers e^- from succinate to ubiquinone, but does not transport protons.
**Nuclear encoded subunits of ubiquinol-cytochrome c reductase (complex III)**	
Uqcr10<br>Uqcr11<br>Uqcrc1<br>Uqcrc2<br>Uqcrh<br>Uqcrfs1<br>Cyc1	Complex III is a multisubunit transmembrane protein encoded by both mitochondrial genes (cytochrome b5) and nuclear genes (all other subunits). Functions to maintain proton gradient across the inner mitochondrial membrane.
**Nuclear encoded subunits of cytochrome c oxidase**	
Cox11<br>Cox7a2<br>Cox6b1<br>Cox5b	Terminal component of MRC. Transfers e^- from cytochrome c to oxygen. Consists of subunits encoded by mitochondrial (e^- transfer) and nuclear (regulation/assembly of complex) genes.
**Nuclear encoded subunits of mitochondrial ATP synthase**	
Atp5k<br>Atp5j2<br>Atp5a1<br>Atp5c1<br>Atp5d	ATP synthase catalyzes the synthesis of ATP, utilizing the electrochemical gradient of protons across the inner mitochondrial membrane. This complex consists of nuclear and mitochondrial encoded genes.

MRC: mitochondrial respiratory chain; e^- : electrons

Figure S2. One-way hierarchical cluster map of oxidative phosphorylation enzymes that changed significantly by at least one diet. mRNA expression of genes that were statistically different from those in mice fed the AIN-93 purified control diet have the percent indicated within the heatmap square (control = 100%).
