# Supplementary material for: Effect of Diet on Expression of Genes Involved in Lipid Metabolism, Oxidative Stress, and Inflammation in Mouse Liver–Insights into Mechanisms of Hepatic Steatosis
Source: PLoS One. 2014 Feb 14;9(2):e88584. doi: 10.1371/journal.pone.0088584 (PMC3925138; doi:10.1371/journal.pone.0088584)
Supplement: Figure S3 — One-way hierarchical cluster map of peroxisomal β-oxidation genes that changed significantly by at least one diet. mRNA expression of genes that were statistically different from those in mice fed the AIN-93 purified control diet have the percent indicated within the heatmap square (control = 100%). (PDF) [file pone.0088584.s003.pdf]

Figure S3

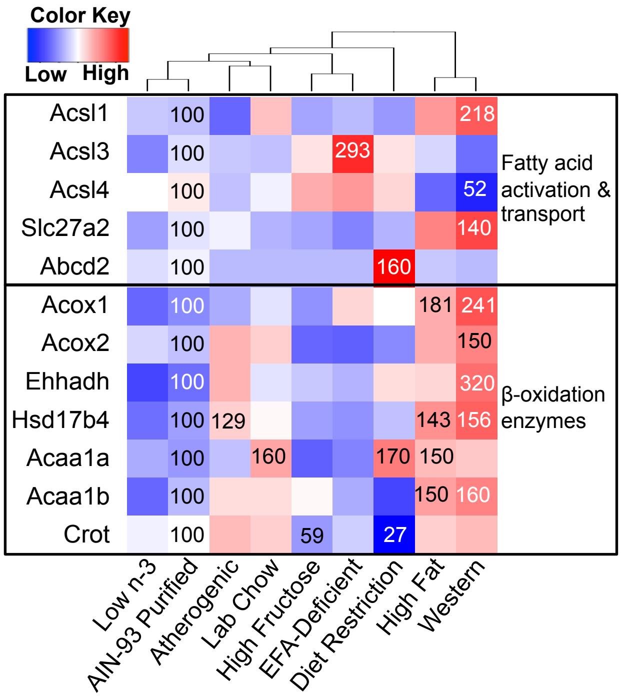

Gene Symbol	Function
**Fatty acid activation and transport**	
Acsl1; 3; 4	Converts free long-chain fatty acids into fatty acyl-CoA esters.
Slc27a2	Converts precursors of bile acids to their CoA thioesters derivatives.
Abcd2	Peroxisomal import of fatty acids and fatty acyl-CoAs.
**Enzymes of peroxisomal β-oxidation**	
Acox1	First enzyme of β-oxidation.
Acox2	Oxidizes the CoA esters of bile acid intermediates.
Ehhadh; Acaa1a; 1b; Hsd17b4	Enzymes of the peroxisomal β-oxidation pathway.
Crot	Converts pristanic acid β-oxidation product into a carnitine ester. This transesterification is necessary for transport of acyl-CoA molecules out of the peroxisome.

Figure S3. One-way hierarchical cluster map of peroxisomal β-oxidation that changed significantly by at least one diet. mRNA expression of genes that were statistically different from those in mice fed the AIN-93 purified control diet have the percent indicated within the heatmap square (control = 100%).
