# Supplementary material for: Effect of Diet on Expression of Genes Involved in Lipid Metabolism, Oxidative Stress, and Inflammation in Mouse Liver–Insights into Mechanisms of Hepatic Steatosis
Source: PLoS One. 2014 Feb 14;9(2):e88584. doi: 10.1371/journal.pone.0088584 (PMC3925138; doi:10.1371/journal.pone.0088584)
Supplement: Table S1 — Gene set enrichment analysis (GSEA). List of all functional gene sets correlated with diet that have an FDR<0.25. (PDF) [file pone.0088584.s004.pdf]

Supplemental Table 1. Gene set enrichment analysis (GSEA). List of all functional gene sets correlated with diet that have an FDR<0.25

Diet	Gene Sets Enriched with FDR < 25%	NES	NOM p-val	FDR q-val
Lab Chow	Gene Sets increased			
	HSA00480_GLUTATHIONE_METABOLISM	2.57	0.000	0.000
	HSA00980_METABOLISM_OF_XENOBIOTICS_BY_CYTOCHROME_P450	2.56	0.000	0.000
	HSA00380_TRYPTOPHAN_METABOLISM	2.31	0.000	0.000
	HSA00280_VALINE_LEUCINE_AND_ISOLEUCINE_DEGRADATION	2.09	0.000	0.002
	HSA00251_GLUTAMATE_METABOLISM	1.89	0.008	0.012
	HSA00350_TYROSINE_METABOLISM	1.77	0.005	0.032
	HSA00071_FATTY_ACID_METABOLISM	1.67	0.01	0.065
	HSA00340_HISTIDINE_METABOLISM	1.63	0.021	0.078
	HSA00252_ALANINE_AND_ASPARTATE_METABOLISM	1.56	0.027	0.108
	HSA00310_LYSINE_DEGRADATION	1.55	0.028	0.105
	Gene Sets Decreased			
	HSA04810_REGULATION_OF_ACTIN_CYTOSKELETON	-1.61	0.01	0.232
High Fructose	Gene Sets Increased			
	HSA00980_METABOLISM_OF_XENOBIOTICS_BY_CYTOCHROME_P450	2.12	0	0.006
	HSA00010_GLYCOLYSIS_AND_GLUCONEOGENESIS	1.96	0	0.023
	HSA00190_OXIDATIVE_PHOSPHORYLATION	1.96	0	0.015
	HSA00020_CITRATE_CYCLE	1.86	0.002	0.026
	HSA04910_INSULIN_SIGNALING_PATHWAY	1.86	0	0.022
	HSA00640_PROPANOATE_METABOLISM	1.79	0	0.032
	HSA00480_GLUTATHIONE_METABOLISM	1.79	0.015	0.03
	HSA00051_FRUCTOSE_AND_MANNOSE_METABOLISM	1.67	0.024	0.067
	HSA05131_PATHOGENIC_ESCHERICHIA_COLI_INFECTION_EPEC	1.65	0.025	0.07
	HSA00620_PYRUVATE_METABOLISM	1.64	0.016	0.069
	HSA00380_TRYPTOPHAN_METABOLISM	1.61	0.011	0.078
	HSA05130_PATHOGENIC_ESCHERICHIA_COLI_INFECTION_EHEC	1.61	0.016	0.072
	HSA00350_TYROSINE_METABOLISM	1.54	0.033	0.098
	HSA00632_BENZOATE_DEGRADATION_VIA_COA_LIGATION	1.5	0.043	0.117
	HSA04920_ADIPOCYTOKINE_SIGNALING_PATHWAY	1.49	0.044	0.116
	HSA03320_PPAR_SIGNALING_PATHWAY	1.43	0.047	0.147
	HSA00071_FATTY_ACID_METABOLISM	1.42	0.072	0.15
	HSA00561_GLYCEROLIPID_METABOLISM	1.39	0.093	0.166
	HSA00650_BUTANOATE_METABOLISM	1.35	0.091	0.19
	HSA00310_LYSINE_DEGRADATION	1.31	0.119	0.229
	Gene Sets Decreased			
	HSA04610_COMPLEMENT_AND_COAGULATION_CASCADES	-1.81	0.002	0.081
	HSA01031_GLYCAN_STRUCTURES_BIOSYNTHESIS_2	-1.77	0.003	0.061
	HSA04060_CYTOKINE_CYTOKINE_RECEPTOR_INTERACTION	-1.67	0.012	0.128
	HSA04620_TOLL_LIKE_RECEPTOR_SIGNALING_PATHWAY	-1.66	0.018	0.1
	HSA04210_APOPTOSIS	-1.48	0.048	0.222

Supplemental Table 1. GSEA output. List of all functional gene sets correlated with diet that have an FDR<0.25

Diet	Gene Sets Enriched with FDR < 25%	NES	NOM p-val	FDR q-val
High Fat	Gene Sets Increased			
	HSA00071_FATTY_ACID_METABOLISM	2.51	0.000	0.000
	HSA00280_VALINE_LEUCINE_AND_ISOLEUCINE_DEGRADATION	2.38	0.000	0.000
	HSA00650_BUTANOATE_METABOLISM	2.25	0.000	0.000
	HSA00980_METABOLISM_OF_XENOBIOTICS_BY_CYTOCHROME_P450	2.24	0.000	0.000
	HSA00310_LYSINE_DEGRADATION	2.23	0.000	0.000
	HSA00100_BIOSYNTHESIS_OF_STEROIDS	2.21	0.000	0.000
	HSA03320_PPAR_SIGNALING_PATHWAY	2.19	0.000	0.000
	HSA00380_TRYPTOPHAN_METABOLISM	2.15	0.000	0.000
	HSA00480_GLUTATHIONE_METABOLISM	2.03	0.000	0.001
	HSA00120_BILE_ACID_BIOSYNTHESIS	1.92	0.004	0.004
	HSA00640_PROPANOATE_METABOLISM	1.91	0.002	0.004
	HSA02010_ABC_TRANSPORTERS_GENERAL	1.83	0.002	0.008
	HSA00561_GLYCEROLIPID_METABOLISM	1.80	0.004	0.009
	HSA00340_HISTIDINE_METABOLISM	1.73	0.007	0.016
	HSA00350_TYROSINE_METABOLISM	1.68	0.016	0.023
	HSA00020_CITRATE_CYCLE	1.53	0.049	0.069
	HSA00010_GLYCOLYSIS_AND_GLUCONEOGENESIS	1.49	0.044	0.081
	HSA00251_GLUTAMATE_METABOLISM	1.48	0.051	0.082
	HSA00252_ALANINE_AND_ASPARTATE_METABOLISM	1.46	0.062	0.090
	HSA04920_ADIPOCYTOKINE_SIGNALING_PATHWAY	1.45	0.066	0.090
	HSA00632_BENZOATE_DEGRADATION_VIA_COA_LIGATION	1.33	0.123	0.176
	Gene Sets Decreased			
	HSA03010_RIBOSOME	-2.30	0.000	0.001
	HSA01031_GLYCAN_STRUCTURES_BIOSYNTHESIS_2	-1.94	0.002	0.029
	HSA04630_JAK_STAT_SIGNALING_PATHWAY	-1.88	0.002	0.033
	HSA04060_CYTOKINE_CYTOKINE_RECEPTOR_INTERACTION	-1.79	0.005	0.062
	HSA05218_MELANOMA	-1.72	0.008	0.078
	HSA01030_GLYCAN_STRUCTURES_BIOSYNTHESIS_1	-1.72	0.007	0.066
	HSA05210_COLORECTAL_CANCER	-1.65	0.016	0.094
	HSA04514_CELL_ADHESION_MOLECULES	-1.62	0.021	0.099
High Saturated Fat & Sucrose	Gene Sets Increased			
	HSA00071_FATTY_ACID_METABOLISM	2.45	0.000	0.000
	HSA00280_VALINE_LEUCINE_AND_ISOLEUCINE_DEGRADATION	2.36	0.000	0.000
	HSA00380_TRYPTOPHAN_METABOLISM	2.28	0.000	0.000
	HSA02010_ABC_TRANSPORTERS_GENERAL	2.26	0.000	0.000
	HSA03320_PPAR_SIGNALING_PATHWAY	2.09	0.000	0.001
	HSA00310_LYSINE_DEGRADATION	1.87	0.004	0.013
	HSA00980_METABOLISM_OF_XENOBIOTICS_BY_CYTOCHROME_P450	1.80	0.002	0.022
	HSA00120_BILE_ACID_BIOSYNTHESIS	1.73	0.012	0.034
	HSA00640_PROPANOATE_METABOLISM	1.72	0.015	0.034
	HSA00480_GLUTATHIONE_METABOLISM	1.70	0.011	0.035
	HSA00650_BUTANOATE_METABOLISM	1.66	0.013	0.042
	HSA00340_HISTIDINE_METABOLISM	1.58	0.030	0.071
	HSA00561_GLYCEROLIPID_METABOLISM	1.45	0.070	0.150
	HSA00632_BENZOATE_DEGRADATION_VIA_COA_LIGATION	1.45	0.066	0.141
	HSA04920_ADIPOCYTOKINE_SIGNALING_PATHWAY	1.44	0.063	0.139
	HSA00350_TYROSINE_METABOLISM	1.33	0.108	0.241
	Gene Sets Decreased			
	HSA00100_BIOSYNTHESIS_OF_STEROIDS	-2.55	0.000	0.000
	HSA03010_RIBOSOME	-2.51	0.000	0.000
	HSA04670_LEUKOCYTE_TRANSENDOTHELIAL_MIGRATION	-1.70	0.013	0.117

Supplemental Table 1. GSEA output. List of all functional gene sets correlated with diet that have an FDR<0.25

Diet	Gene Sets Enriched with FDR < 25%	NES	NOM p-val	FDR q-val
High Cholesterol & CA	Gene Sets Increased			
	HSA04612_ANTIGEN_PROCESSING_AND_PRESENTATION	2.04	0	0.003
	HSA00980_METABOLISM_OF_XENOBIOTICS_BY_CYTOCHROME_P450	2.03	0	0.001
	HSA00480_GLUTATHIONE_METABOLISM	1.96	0	0.002
	HSA02010_ABC_TRANSPORTERS_GENERAL	1.93	0	0.002
	HSA04514_CELL_ADHESION_MOLECULES	1.81	0.003	0.015
	HSA00340_HISTIDINE_METABOLISM	1.69	0.011	0.048
	Gene Sets Decreased			
	HSA00100_BIOSYNTHESIS_OF_STEROIDS	-2.74	0	0
	HSA03010_RIBOSOME	-2.43	0	0
	HSA00260_GLYCINE_SERINE_AND_THREONINE_METABOLISM	-1.72	0.017	0.09
	HSA00190_OXIDATIVE_PHOSPHORYLATION	-1.62	0.014	0.143
EFA Deficient	Gene Sets Increased			
	HSA00100_BIOSYNTHESIS_OF_STEROIDS	1.97	0	0.014
	HSA00020_CITRATE_CYCLE	1.78	0.005	0.091
	HSA00350_TYROSINE_METABOLISM	1.69	0.003	0.141
	HSA05130_PATHOGENIC_ESCHERICHIA_COLI_INFECTION_EHEC	1.65	0.02	0.156
	HSA05131_PATHOGENIC_ESCHERICHIA_COLI_INFECTION_EPEC	1.65	0.025	0.127
	HSA00640_PROPANOATE_METABOLISM	1.59	0.022	0.173
	HSA03320_PPAR_SIGNALING_PATHWAY	1.58	0.01	0.157
	HSA00071_FATTY_ACID_METABOLISM	1.55	0.018	0.171
	HSA05215_PROSTATE_CANCER	1.5	0.037	0.222
	Gene Sets Decreased			
	HSA04610_COMPLEMENT_AND_COAGULATION_CASCADES	-1.94	0	0.013
	HSA03010_RIBOSOME	-1.9	0	0.01
	HSA04060_CYTOKINE_CYTOKINE_RECEPTOR_INTERACTION	-1.79	0.007	0.03
	HSA01031_GLYCAN_STRUCTURES_BIOSYNTHESIS_2	-1.78	0.002	0.025
DHA Deficient	Gene Sets Increased			
	HSA00980_METABOLISM_OF_XENOBIOTICS_BY_CYTOCHROME_P450	2	0.003	0.019
	HSA00190_OXIDATIVE_PHOSPHORYLATION	1.95	0.004	0.017
	HSA00480_GLUTATHIONE_METABOLISM	1.77	0.003	0.066
	HSA05213_ENDOMETRIAL_CANCER	1.66	0.015	0.115
	HSA05215_PROSTATE_CANCER	1.59	0.003	0.149
	HSA00251_GLUTAMATE_METABOLISM	1.56	0.026	0.15
	HSA05130_PATHOGENIC_ESCHERICHIA_COLI_INFECTION_EHEC	1.51	0.06	0.183
	HSA05131_PATHOGENIC_ESCHERICHIA_COLI_INFECTION_EPEC	1.49	0.046	0.188
	HSA00350_TYROSINE_METABOLISM	1.43	0.065	0.233
	Gene Sets Decreased			
	Non with FDR<25%			

Supplemental Table 1. GSEA output. List of all functional gene sets correlated with diet that have an FDR<0.25

GS	Gene Sets Enriched with FDR < 25%			
Diet Restriction UPREGULATED	Gene Set	NES	NOM p-val	FDR q-val
	HSA00980_METABOLISM_OF_XENOBIOTICS_BY_CYTOCHROME_P450	2.54	0	0
	HSA00480_GLUTATHIONE_METABOLISM	2.31	0	0
	HSA04920_ADIPOCYTOKINE_SIGNALING_PATHWAY	2.13	0	0.001
	HSA02010_ABC_TRANSPORTERS_GENERAL	1.78	0.01	0.039
	HSA00280_VALINE_LEUCINE_AND_ISOLEUCINE_DEGRADATION	1.64	0.012	0.109
	HSA00190_OXIDATIVE_PHOSPHORYLATION	1.55	0.022	0.178
	HSA03010_RIBOSOME	1.49	0.038	0.219
Diet Restriction DOWNREGULATED	Gene Set	NES	NOM p-val	FDR q-val
	HSA04060_CYTOKINE_CYTOKINE_RECEPTOR_INTERACTION	-1.59	0.03	0.249
	HSA04530_TIGHT_JUNCTION	-1.57	0.034	0.235
	HSA04115_P53_SIGNALING_PATHWAY	-1.54	0.024	0.246
	HSA01031_GLYCAN_STRUCTURES_BIOSYNTHESIS_2	-1.51	0.04	0.246
	HSA01030_GLYCAN_STRUCTURES_BIOSYNTHESIS_1	-1.5	0.043	0.244
	HSA00100_BIOSYNTHESIS_OF_STEROIDS	-1.48	0.073	0.242
	HSA04670_LEUKOCYTE_TRANSENDOTHELIAL_MIGRATION	-1.43	0.066	0.249
	HSA05131_PATHOGENIC_ESCHERICHIA_COLI_INFECTION_EPEC	-1.42	0.074	0.247
