# Supplementary material for: Effect of Diet on Expression of Genes Involved in Lipid Metabolism, Oxidative Stress, and Inflammation in Mouse Liver–Insights into Mechanisms of Hepatic Steatosis
Source: PLoS One. 2014 Feb 14;9(2):e88584. doi: 10.1371/journal.pone.0088584 (PMC3925138; doi:10.1371/journal.pone.0088584)
Supplement: Table S2 — The effect of diet on the rate limiting step or biomarker of key pathways. (PDF) [file pone.0088584.s005.pdf]

Table S2. The effect of diet on the rate limiting step or biomarker of key pathways.

Pathway	Rate limiting	Diet
Fatty acid biosynthesis	Acaca	Increased in:<br>• High Fructose, EFA-Deficient<br>Decreased in:<br>• Lab chow
Mitochondrial β-oxidation	Cpt1a	Increased in:<br>• High Fat
Cholesterol biosynthesis	Hmgcr	Decreased in:<br>• Western, Atherogenic, Low n-3
Glutathione metabolism	Gclc	Increased in:<br>• Western, Atherogenic, Lab chow<br>Decreased in:<br>• Diet restriction
Marker of oxidative stress	Gpx1	Increased in:<br>• High Fat, Western, Atherogenic, Lab chow
Peroxisomal β-oxidation	Acox1	Increased in:<br>• High Fat, Western
Marker of inflammation	Icam1	Increased in:<br>• Atherogenic
