# Supplementary material for: Effect of Diet on Expression of Genes Involved in Lipid Metabolism, Oxidative Stress, and Inflammation in Mouse Liver–Insights into Mechanisms of Hepatic Steatosis
Source: PLoS One. 2014 Feb 14;9(2):e88584. doi: 10.1371/journal.pone.0088584 (PMC3925138; doi:10.1371/journal.pone.0088584)
Supplement: Table S3 — Oligonucleotide primers used for qPCR. (PDF) [file pone.0088584.s006.pdf]

Table S3. Oligonucleotide primers used for qPCR

Name	GenBank Accession No.	Direction	Primer Sequence
Abcb1a	NM_011076	F	GCGACTCCGATACATGGTTT
		R	ACCCTGTAGCCCCTTTCACT
Cyp7a1	NM_007824	F	ATCCTGGCAAACAGAAATCG
		R	GGCCAAGTCTGGTTTCTCTG
Fabp5	NM_010634.3	F	CAAAACCGAGAGCACAGTGA
		R	CACGATCATCTTCCCATCCT
Elovl3	NM_007703.2	F	ATGAACTTTGGCGTCCATTC
		R	CTTTCTCCTGCCTCCAGATG
Vldlr	NM_001161420.1	F	TCCTGATTGCGAAGACGGTT
	and NM_013703.2	R	GCGCCACAGCTGATTTCATT
CFD	NM_013459.2	F	TCGAAGGTGTGGTTACGTGG
		R	TCGATCCACATCCGGTAGGA
C6	NM_016704.2	F	GTGACAGTGGGCGCTGTATT
		R	GGCTTTGTCCTCCCACAGTT
C9	NM_013485.1	F	AGGGACTAGGGAGCAAGCAA
		R	CCCAGTTGGCGAAGTCAGTC
C8a	NM_146148.2	F	GGTGGACTTGGAAGGGTCAA
		R	GTGACTGGCTCTTTTCGGGA
C8b	NM_133882.2	F	CTGGCAAAGTCGTGTCTCCT
		R	ACTCCGTACTGGTCTGCTCT
C8g	NM_001271777.1	F	ATTGCATGCATAGGGTTGAGC
		R	ATAGGAGCTGGGCATTGTGT
Icam1	NM_010493.2	F	GTCTCGGAAGGGAGCCAAGTA
		R	TATCCGAGCTTCAGAGGCAG
Gsta4	NM_010357	F	AGTGCAGCGTGCTTTAAGGT
		R	GGGCAGAGTGGTTTTGTTGT
